# 7,14-Bis(4-methoxy­phen­yl)-11,11-dimethyl-1,4,10,12-tetra­oxa­dispiro­[4.2.5.2]penta­decane-9,13-dione

**DOI:** 10.1107/S1600536809013658

**Published:** 2009-04-18

**Authors:** Jinpeng Zhang, Shu Yan, Jie Ding

**Affiliations:** a Department of Public Health, Xuzhou Medical College, Xuzhou 221000, People’s Republic of China; b College of Chemistry and Chemical Engineering, Xuzhou Normal University, Xuzhou 221116, People’s Republic of China

## Abstract

In the title compound, C_27_H_30_O_8_, the cyclo­hexane ring is in a chair conformation, while the five-membered ring adopts an envelope conformation. The 1,3-dioxane ring is oriented with respect to the benzene rings at dihedral angles of 53.38 (3) and 55.31 (3)°, while the dihedral angle between the benzene rings is 71.56 (3)°. In the crystal structure, inter­molecular C—H⋯O inter­actions link the mol­ecules into chains.

## Related literature

For general background on Meldrum’s acid, see: Davidson & Bernhard (1948 ▶); Meldrum (1908 ▶); Muller *et al.* (2005 ▶); Ramachary *et al.* (2003 ▶); Tietze & Beifuss (1993 ▶); Tietze *et al.* (2001 ▶). For related structures, see: Chande & Khanwelkar (2005 ▶); Ramachary & Barbas (2004 ▶). For bond-length data, see: Allen *et al.* (1987 ▶). For ring-puckering parameters, see: Cremer & Pople (1975 ▶).
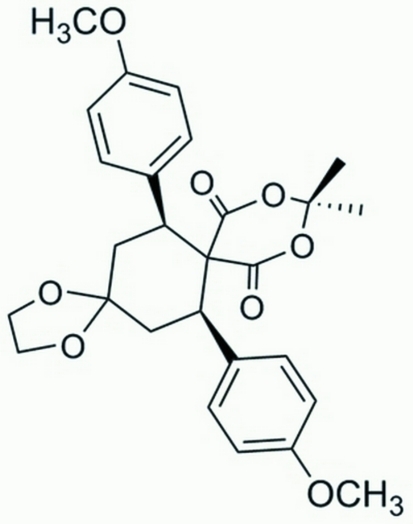


## Experimental

### 

#### Crystal data

C_27_H_30_O_8_
*M* *_r_* = 482.51
Monoclinic, 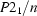

*a* = 9.977 (5) Å
*b* = 20.162 (9) Å
*c* = 12.508 (6) Å
β = 94.934 (8)°
*V* = 2507 (2) Å^3^
*Z* = 4
Mo *K*α radiation
μ = 0.09 mm^−1^
*T* = 298 K
0.43 × 0.25 × 0.12 mm

#### Data collection

Bruker SMART CCD area-detector diffractometer
Absorption correction: multi-scan (*SADABS*; Sheldrick, 1996 ▶) *T*_min_ = 0.961, *T*_max_ = 0.989
11754 measured reflections
4126 independent reflections
1349 reflections with *I* > 2σ(*I*)
*R*_int_ = 0.104

#### Refinement

*R*[*F* ^2^ > 2σ(*F* ^2^)] = 0.075
*wR*(*F* ^2^) = 0.187
*S* = 1.01
4126 reflections
316 parameters
H-atom parameters constrained
Δρ_max_ = 0.46 e Å^−3^
Δρ_min_ = −0.21 e Å^−3^

### 

Data collection: *SMART* (Bruker, 1998 ▶); cell refinement: *SAINT* (Bruker, 1998 ▶); data reduction: *SAINT*; program(s) used to solve structure: *SHELXS97* (Sheldrick, 2008 ▶); program(s) used to refine structure: *SHELXL97* (Sheldrick, 2008 ▶); molecular graphics: *ORTEP-3 for Windows* (Farrugia, 1997 ▶) and *PLATON* (Spek, 2009 ▶); software used to prepare material for publication: *SHELXL97*.

## Supplementary Material

Crystal structure: contains datablocks global, I. DOI: 10.1107/S1600536809013658/hk2665sup1.cif

Structure factors: contains datablocks I. DOI: 10.1107/S1600536809013658/hk2665Isup2.hkl

Additional supplementary materials:  crystallographic information; 3D view; checkCIF report

## Figures and Tables

**Table 1:** Hydrogen-bond geometry (Å, °)

*D*—H⋯*A*	*D*—H	H⋯*A*	*D*⋯*A*	*D*—H⋯*A*
C16—H16⋯O4^i^	0.93	2.53	3.449 (3)	168

Symmetry code: (i) 
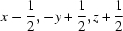
.

**Fig. 1.**
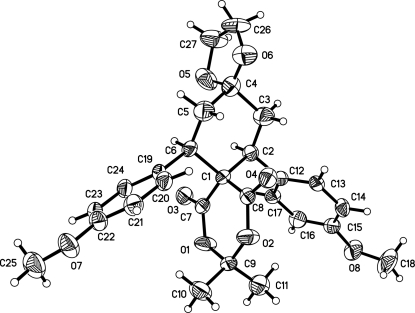
The molecular structure of the title molecule, with the atom-numbering scheme. Displacement ellipsoids are drawn at the 30% probability level.

**Fig. 2.**
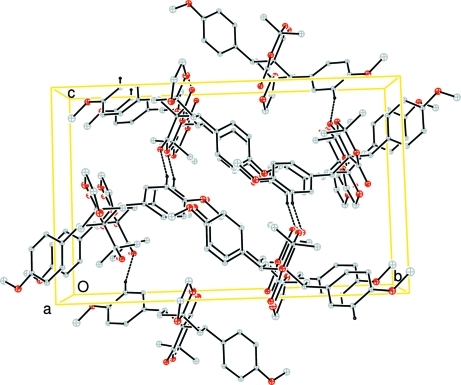
A partial packing diagram of the title compound. Hydrogen bonds are shown as dashed lines.

## supplementary crystallographic information

### Comment

Over the past few decades, Meldrum's acid (2,2-dimethyl-1,3-dioxane-4,6-dione) (Tietze & Beifuss, 1993; Tietze *et al.*,  2001) has been used as a versatile organic reagent (Ramachary *et al.*,  2003; Muller *et al.*, 2005) and its derivatives are very useful building blocks in synthetic organic chemistry (Davidson & Bernhard, 1948; Meldrum, 1908). Spirocyclic compounds including a Meldrum's acid unit are attractive intermediates in the syntheses of natural products and in medicinal chemistry. Thus, the synthesis of new highly substituted spiro ring system with a Meldrum's acid unit has attracted widespread attention (Ramachary & Barbas, 2004; Chande & Khanwelkar, 2005). We report herein the crystal structure of the title compound.

In the molecule of the title compound (Fig. 1), the bond lengths (Allen *et al.*,  1987) and angles are within normal ranges. Ring A (C1-C6) is not planar, having total puckering amplitude, Q_T_, of 0.500 (2) Å and chair conformation [φ = 7.64 (3) and θ = 175.56 (3) °] (Cremer & Pople, 1975). Rings B (O1/O2/C1/C7-C9), D (C12-C17) and E (C19-C24) are, of course, planar, and they are oriented at dihedral angles of B/D = 53.38 (3), B/E = 55.31 (3) and D/E = 71.56 (3) °. Ring C (O5/O6/C4/C26/C27) adopts envelope conformation, with atom O5 displaced by 0.153 (3) Å from the plane of the other ring atoms.

In the crystal structure, intermolecular C-H···O interactions (Table 1) link the molecules into chains (Fig. 2), in which they may be effective in the stabilization of the structure.

### Experimental

The title compound was prepared by the reaction of of 1,2-diarylidenehydrazine (2 mmol), Meldrum's acid (5 mmol), HOAc (4 ml) and and ethane-1,2-diol (8 ml).

### Refinement

H atoms were positioned geometrically, with C-H = 0.93, 0.98, 0.97 and 0.96 Å for aromatic, methine, methylene and methyl H, respectively, and constrained to ride on their parent atoms, with U_iso_(H) = xU_eq_(C), where x = 1.5 for methyl H and x = 1.2 for all other H atoms.

### Crystal data

**Table:**

C_27_H_30_O_8_	*F*(000) = 1024
*M**_r_* = 482.51	*D*_x_ = 1.279 Mg m^−^^3^
Monoclinic, *P*2_1_/*n*	Melting point: 479 K
Hall symbol: -P 2yn	Mo *K*α radiation, λ = 0.71073 Å
*a* = 9.977 (5) Å	Cell parameters from 1366 reflections
*b* = 20.162 (9) Å	θ = 2.3–19.5°
*c* = 12.508 (6) Å	µ = 0.09 mm^−^^1^
β = 94.934 (8)°	*T* = 298 K
*V* = 2507 (2) Å^3^	Prism, colorless
*Z* = 4	0.43 × 0.25 × 0.12 mm

### Data collection

**Table:**

Bruker SMART CCD area-detector diffractometer	4126 independent reflections
Radiation source: fine-focus sealed tube	1349 reflections with *I* > 2σ(*I*)
graphite	*R*_int_ = 0.104
φ and ω scans	θ_max_ = 25.0°, θ_min_ = 2.3°
Absorption correction: multi-scan (*SADABS*; Sheldrick, 1996)	*h* = −11→11
*T*_min_ = 0.961, *T*_max_ = 0.989	*k* = −20→24
11754 measured reflections	*l* = −14→14

### Refinement

**Table:**

Refinement on *F*^2^	Secondary atom site location: difference Fourier map
Least-squares matrix: full	Hydrogen site location: inferred from neighbouring sites
*R*[*F*^2^ > 2σ(*F*^2^)] = 0.075	H-atom parameters constrained
*wR*(*F*^2^) = 0.187	*w* = 1/[σ^2^(*F*_o_^2^) + (0.055*P*)^2^] where *P* = (*F*_o_^2^ + 2*F*_c_^2^)/3
*S* = 1.00	(Δ/σ)_max_ < 0.001
4126 reflections	Δρ_max_ = 0.46 e Å^−^^3^
316 parameters	Δρ_min_ = −0.21 e Å^−^^3^
Primary atom site location: structure-invariant direct methods	

### Special details

**Table:**

Geometry. All e.s.d.'s (except the e.s.d. in the dihedral angle between two l.s. planes) are estimated using the full covariance matrix. The cell e.s.d.'s are taken into account individually in the estimation of e.s.d.'s in distances, angles and torsion angles; correlations between e.s.d.'s in cell parameters are only used when they are defined by crystal symmetry. An approximate (isotropic) treatment of cell e.s.d.'s is used for estimating e.s.d.'s involving l.s. planes.
Refinement. Refinement of *F*^2^ against ALL reflections. The weighted *R*-factor *wR* and goodness of fit *S* are based on *F*^2^, conventional *R*-factors *R* are based on *F*, with *F* set to zero for negative *F*^2^. The threshold expression of *F*^2^ > σ(*F*^2^) is used only for calculating *R*-factors(gt) *etc*. and is not relevant to the choice of reflections for refinement. *R*-factors based on *F*^2^ are statistically about twice as large as those based on *F*, and *R*- factors based on ALL data will be even larger.

### Fractional atomic coordinates and isotropic or equivalent isotropic displacement parameters (Å^2^)

**Table:**

	*x*	*y*	*z*	*U*_iso_*/*U*_eq_	
O1	−0.0782 (3)	0.1365 (2)	0.3511 (3)	0.0761 (13)	
O2	0.0250 (4)	0.1844 (2)	0.2024 (3)	0.0751 (12)	
O3	0.0355 (4)	0.10348 (19)	0.4940 (3)	0.0686 (12)	
O4	0.2363 (4)	0.19884 (18)	0.2038 (3)	0.0649 (11)	
O5	0.4608 (4)	0.1128 (2)	0.5381 (4)	0.1049 (16)	
O6	0.5876 (5)	0.1554 (2)	0.4129 (4)	0.1001 (16)	
O7	−0.0022 (5)	−0.1063 (2)	0.0771 (3)	0.0887 (14)	
O8	−0.1002 (4)	0.4239 (2)	0.4598 (4)	0.0898 (14)	
C1	0.1680 (5)	0.1482 (2)	0.3627 (4)	0.0466 (14)	
C2	0.2380 (5)	0.1994 (3)	0.4430 (5)	0.0615 (16)	
H2	0.2350	0.1788	0.5137	0.074*	
C3	0.3789 (6)	0.2090 (3)	0.4312 (5)	0.082 (2)	
H3A	0.4153	0.2374	0.4892	0.099*	
H3B	0.3872	0.2327	0.3646	0.099*	
C4	0.4711 (8)	0.1438 (3)	0.4308 (5)	0.0680 (18)	
C5	0.3969 (6)	0.0959 (3)	0.3462 (6)	0.084 (2)	
H5A	0.4050	0.1141	0.2753	0.101*	
H5B	0.4438	0.0537	0.3498	0.101*	
C6	0.2578 (5)	0.0834 (3)	0.3569 (5)	0.0588 (16)	
H6	0.2569	0.0637	0.4284	0.071*	
C7	0.0383 (5)	0.1274 (3)	0.4064 (5)	0.0528 (15)	
C8	0.1480 (6)	0.1791 (2)	0.2534 (5)	0.0475 (14)	
C9	−0.0965 (5)	0.1675 (3)	0.2462 (4)	0.0507 (14)	
C10	−0.1679 (6)	0.1184 (3)	0.1731 (5)	0.091 (2)	
H10A	−0.2520	0.1068	0.2002	0.137*	
H10B	−0.1134	0.0794	0.1692	0.137*	
H10C	−0.1845	0.1374	0.1029	0.137*	
C11	−0.1733 (6)	0.2284 (3)	0.2604 (5)	0.090 (2)	
H11A	−0.2563	0.2176	0.2898	0.135*	
H11B	−0.1922	0.2498	0.1922	0.135*	
H11C	−0.1217	0.2577	0.3084	0.135*	
C12	0.1543 (5)	0.2613 (3)	0.4480 (5)	0.0537 (15)	
C13	0.1714 (5)	0.3153 (3)	0.3840 (5)	0.0556 (15)	
H13	0.2404	0.3148	0.3385	0.067*	
C14	0.0903 (6)	0.3702 (3)	0.3850 (5)	0.0606 (16)	
H14	0.1028	0.4054	0.3389	0.073*	
C15	−0.0101 (6)	0.3731 (3)	0.4544 (5)	0.0615 (16)	
C16	−0.0247 (6)	0.3210 (3)	0.5229 (5)	0.0660 (17)	
H16	−0.0905	0.3227	0.5712	0.079*	
C17	0.0572 (5)	0.2659 (3)	0.5209 (4)	0.0560 (15)	
H17	0.0472	0.2314	0.5689	0.067*	
C18	−0.1000 (8)	0.4737 (4)	0.3811 (7)	0.134 (3)	
H18A	−0.1667	0.5064	0.3936	0.201*	
H18B	−0.1202	0.4544	0.3114	0.201*	
H18C	−0.0129	0.4942	0.3844	0.201*	
C19	0.1895 (5)	0.0320 (3)	0.2828 (5)	0.0516 (15)	
C20	0.2011 (5)	0.0315 (3)	0.1736 (5)	0.0571 (15)	
H20	0.2529	0.0640	0.1441	0.069*	
C21	0.1380 (6)	−0.0158 (3)	0.1067 (5)	0.0685 (17)	
H21	0.1504	−0.0159	0.0339	0.082*	
C22	0.0572 (6)	−0.0626 (3)	0.1485 (6)	0.0643 (17)	
C23	0.0439 (5)	−0.0631 (3)	0.2572 (6)	0.0610 (16)	
H23	−0.0086	−0.0955	0.2861	0.073*	
C24	0.1071 (5)	−0.0163 (3)	0.3232 (5)	0.0595 (15)	
H24	0.0949	−0.0167	0.3960	0.071*	
C25	−0.0945 (7)	−0.1527 (3)	0.1126 (6)	0.108 (3)	
H25A	−0.1280	−0.1801	0.0535	0.162*	
H25B	−0.1680	−0.1296	0.1405	0.162*	
H25C	−0.0502	−0.1799	0.1678	0.162*	
C26	0.6675 (6)	0.1443 (4)	0.5066 (6)	0.102 (3)	
H26A	0.7119	0.1854	0.5290	0.123*	
H26B	0.7367	0.1124	0.4926	0.123*	
C27	0.5964 (7)	0.1206 (4)	0.5904 (6)	0.115 (3)	
H27A	0.5981	0.1523	0.6489	0.138*	
H27B	0.6324	0.0786	0.6176	0.138*	

### Atomic displacement parameters (Å^2^)

**Table:**

	*U*^11^	*U*^22^	*U*^33^	*U*^12^	*U*^13^	*U*^23^
O1	0.037 (2)	0.115 (4)	0.076 (3)	−0.008 (2)	0.002 (2)	0.022 (3)
O2	0.049 (2)	0.113 (4)	0.062 (3)	−0.004 (2)	−0.003 (2)	0.026 (2)
O3	0.076 (3)	0.070 (3)	0.059 (3)	−0.008 (2)	0.000 (2)	0.015 (2)
O4	0.065 (2)	0.064 (3)	0.069 (3)	0.004 (2)	0.023 (2)	0.009 (2)
O5	0.074 (3)	0.096 (4)	0.152 (4)	−0.018 (3)	0.050 (3)	−0.033 (3)
O6	0.082 (3)	0.115 (4)	0.101 (4)	0.007 (3)	−0.004 (3)	−0.004 (3)
O7	0.100 (3)	0.075 (3)	0.088 (3)	−0.023 (3)	−0.005 (3)	−0.022 (3)
O8	0.082 (3)	0.064 (3)	0.127 (4)	0.031 (3)	0.030 (3)	−0.002 (3)
C1	0.043 (3)	0.043 (3)	0.054 (4)	0.007 (3)	0.001 (3)	0.001 (3)
C2	0.064 (4)	0.044 (4)	0.073 (4)	0.002 (3)	−0.014 (3)	−0.017 (3)
C3	0.067 (4)	0.091 (5)	0.089 (5)	0.005 (4)	0.010 (4)	−0.004 (4)
C4	0.080 (5)	0.072 (5)	0.054 (4)	0.006 (4)	0.016 (4)	0.009 (4)
C5	0.068 (4)	0.082 (5)	0.106 (6)	0.008 (4)	0.024 (4)	0.004 (4)
C6	0.058 (4)	0.038 (4)	0.078 (4)	0.012 (3)	−0.008 (3)	−0.012 (3)
C7	0.048 (4)	0.041 (4)	0.070 (4)	0.009 (3)	0.009 (3)	−0.006 (3)
C8	0.048 (3)	0.040 (3)	0.057 (4)	0.003 (3)	0.013 (3)	−0.010 (3)
C9	0.047 (3)	0.056 (4)	0.047 (4)	0.002 (3)	−0.008 (3)	0.006 (3)
C10	0.069 (4)	0.089 (5)	0.113 (6)	−0.012 (4)	−0.012 (4)	−0.024 (4)
C11	0.085 (5)	0.078 (5)	0.105 (6)	0.006 (4)	0.000 (4)	−0.008 (4)
C12	0.049 (3)	0.042 (4)	0.068 (4)	0.004 (3)	−0.004 (3)	−0.012 (3)
C13	0.051 (3)	0.046 (4)	0.069 (4)	−0.006 (3)	−0.004 (3)	−0.008 (3)
C14	0.068 (4)	0.041 (4)	0.074 (4)	−0.004 (3)	0.015 (3)	−0.008 (3)
C15	0.056 (4)	0.048 (4)	0.078 (5)	0.008 (3)	−0.002 (3)	−0.018 (4)
C16	0.065 (4)	0.062 (5)	0.072 (5)	0.006 (4)	0.010 (3)	−0.021 (4)
C17	0.049 (3)	0.057 (4)	0.063 (4)	0.004 (3)	0.006 (3)	−0.002 (3)
C18	0.145 (7)	0.078 (6)	0.182 (9)	0.055 (6)	0.032 (6)	0.026 (6)
C19	0.049 (3)	0.033 (4)	0.073 (5)	0.002 (3)	0.004 (3)	−0.003 (3)
C20	0.060 (4)	0.047 (4)	0.066 (4)	0.003 (3)	0.012 (3)	−0.001 (3)
C21	0.078 (4)	0.058 (4)	0.069 (4)	0.005 (4)	0.003 (4)	−0.016 (4)
C22	0.068 (4)	0.055 (5)	0.068 (5)	−0.001 (4)	−0.002 (4)	−0.020 (4)
C23	0.058 (4)	0.043 (4)	0.081 (5)	−0.003 (3)	0.002 (4)	0.012 (3)
C24	0.071 (4)	0.042 (4)	0.064 (4)	0.001 (3)	−0.004 (3)	−0.002 (3)
C25	0.106 (6)	0.085 (6)	0.133 (7)	−0.011 (5)	0.009 (5)	−0.044 (5)
C26	0.051 (4)	0.174 (8)	0.077 (5)	0.012 (5)	−0.021 (4)	0.000 (5)
C27	0.067 (5)	0.185 (9)	0.091 (6)	−0.014 (5)	−0.008 (4)	0.035 (5)

### Geometric parameters (Å, °)

**Table:**

O1—C7	1.313 (6)	C11—H11A	0.9600
O1—C9	1.451 (6)	C11—H11B	0.9600
O2—C8	1.338 (6)	C11—H11C	0.9600
O2—C9	1.415 (6)	C12—C13	1.371 (7)
O3—C7	1.199 (6)	C12—C17	1.389 (7)
O4—C8	1.188 (5)	C13—C14	1.371 (7)
O5—C27	1.460 (7)	C13—H13	0.9300
O5—C4	1.492 (7)	C14—C15	1.382 (7)
O6—C4	1.225 (7)	C14—H14	0.9300
O6—C26	1.377 (7)	C15—C16	1.372 (8)
O7—C22	1.353 (6)	C16—C17	1.380 (7)
O7—C25	1.410 (7)	C16—H16	0.9300
O8—C15	1.368 (6)	C17—H17	0.9300
O8—C18	1.406 (8)	C18—H18A	0.9600
C1—C8	1.500 (7)	C18—H18B	0.9600
C1—C7	1.507 (7)	C18—H18C	0.9600
C1—C2	1.562 (7)	C19—C20	1.380 (7)
C1—C6	1.590 (6)	C19—C24	1.397 (7)
C2—C3	1.440 (7)	C20—C21	1.384 (7)
C2—C12	1.506 (7)	C20—H20	0.9300
C2—H2	0.9800	C21—C22	1.374 (7)
C3—C4	1.605 (8)	C21—H21	0.9300
C3—H3A	0.9700	C22—C23	1.378 (7)
C3—H3B	0.9700	C23—C24	1.372 (7)
C4—C5	1.570 (8)	C23—H23	0.9300
C5—C6	1.428 (7)	C24—H24	0.9300
C5—H5A	0.9700	C25—H25A	0.9600
C5—H5B	0.9700	C25—H25B	0.9600
C6—C19	1.512 (7)	C25—H25C	0.9600
C6—H6	0.9800	C26—C27	1.400 (8)
C9—C11	1.466 (7)	C26—H26A	0.9700
C9—C10	1.486 (7)	C26—H26B	0.9700
C10—H10A	0.9600	C27—H27A	0.9700
C10—H10B	0.9600	C27—H27B	0.9700
C10—H10C	0.9600		
			
C7—O1—C9	125.0 (4)	C9—C11—H11C	109.5
C8—O2—C9	125.3 (4)	H11A—C11—H11C	109.5
C27—O5—C4	103.0 (5)	H11B—C11—H11C	109.5
C4—O6—C26	107.6 (6)	C13—C12—C17	117.3 (5)
C22—O7—C25	119.0 (5)	C13—C12—C2	122.5 (5)
C15—O8—C18	117.3 (5)	C17—C12—C2	120.2 (6)
C8—C1—C7	113.3 (4)	C12—C13—C14	122.2 (5)
C8—C1—C2	109.2 (4)	C12—C13—H13	118.9
C7—C1—C2	107.5 (4)	C14—C13—H13	118.9
C8—C1—C6	109.3 (4)	C13—C14—C15	120.0 (6)
C7—C1—C6	107.0 (4)	C13—C14—H14	120.0
C2—C1—C6	110.5 (4)	C15—C14—H14	120.0
C3—C2—C12	116.3 (5)	O8—C15—C16	115.9 (6)
C3—C2—C1	114.2 (5)	O8—C15—C14	125.3 (6)
C12—C2—C1	110.8 (4)	C16—C15—C14	118.8 (6)
C3—C2—H2	104.7	C15—C16—C17	120.7 (6)
C12—C2—H2	104.7	C15—C16—H16	119.7
C1—C2—H2	104.7	C17—C16—H16	119.7
C2—C3—C4	117.1 (6)	C16—C17—C12	120.9 (6)
C2—C3—H3A	108.0	C16—C17—H17	119.6
C4—C3—H3A	108.0	C12—C17—H17	119.6
C2—C3—H3B	108.0	O8—C18—H18A	109.5
C4—C3—H3B	108.0	O8—C18—H18B	109.5
H3A—C3—H3B	107.3	H18A—C18—H18B	109.5
O6—C4—O5	112.6 (6)	O8—C18—H18C	109.5
O6—C4—C5	113.2 (6)	H18A—C18—H18C	109.5
O5—C4—C5	106.5 (5)	H18B—C18—H18C	109.5
O6—C4—C3	113.4 (6)	C20—C19—C24	117.1 (5)
O5—C4—C3	104.8 (5)	C20—C19—C6	122.6 (5)
C5—C4—C3	105.6 (5)	C24—C19—C6	120.3 (6)
C6—C5—C4	116.8 (5)	C19—C20—C21	122.0 (6)
C6—C5—H5A	108.1	C19—C20—H20	119.0
C4—C5—H5A	108.1	C21—C20—H20	119.0
C6—C5—H5B	108.1	C22—C21—C20	119.7 (6)
C4—C5—H5B	108.1	C22—C21—H21	120.1
H5A—C5—H5B	107.3	C20—C21—H21	120.1
C5—C6—C19	117.0 (5)	O7—C22—C21	115.8 (6)
C5—C6—C1	114.6 (5)	O7—C22—C23	124.9 (6)
C19—C6—C1	111.4 (4)	C21—C22—C23	119.3 (6)
C5—C6—H6	104.0	C24—C23—C22	120.7 (6)
C19—C6—H6	104.0	C24—C23—H23	119.6
C1—C6—H6	104.0	C22—C23—H23	119.6
O3—C7—O1	116.7 (5)	C23—C24—C19	121.1 (6)
O3—C7—C1	122.1 (5)	C23—C24—H24	119.4
O1—C7—C1	121.2 (5)	C19—C24—H24	119.4
O4—C8—O2	114.5 (5)	O7—C25—H25A	109.5
O4—C8—C1	124.6 (5)	O7—C25—H25B	109.5
O2—C8—C1	120.8 (5)	H25A—C25—H25B	109.5
O2—C9—O1	114.1 (4)	O7—C25—H25C	109.5
O2—C9—C11	108.6 (5)	H25A—C25—H25C	109.5
O1—C9—C11	106.1 (5)	H25B—C25—H25C	109.5
O2—C9—C10	107.7 (5)	O6—C26—C27	113.5 (6)
O1—C9—C10	106.6 (5)	O6—C26—H26A	108.9
C11—C9—C10	113.8 (5)	C27—C26—H26A	108.9
C9—C10—H10A	109.5	O6—C26—H26B	108.9
C9—C10—H10B	109.5	C27—C26—H26B	108.9
H10A—C10—H10B	109.5	H26A—C26—H26B	107.7
C9—C10—H10C	109.5	C26—C27—O5	102.1 (5)
H10A—C10—H10C	109.5	C26—C27—H27A	111.3
H10B—C10—H10C	109.5	O5—C27—H27A	111.3
C9—C11—H11A	109.5	C26—C27—H27B	111.3
C9—C11—H11B	109.5	O5—C27—H27B	111.3
H11A—C11—H11B	109.5	H27A—C27—H27B	109.2

### Hydrogen-bond geometry (Å, °)

**Table:**

*D*—H···*A*	*D*—H	H···*A*	*D*···*A*	*D*—H···*A*
C16—H16···O4^i^	0.93	2.53	3.449 (3)	168

Symmetry codes: (i) *x*−1/2, −*y*+1/2, *z*+1/2.

## References

[bb1] Allen, F. H., Kennard, O., Watson, D. G., Brammer, L., Orpen, A. G. & Taylor, R. (1987). *J. Chem. Soc. Perkin Trans. 2*, pp. S1–19.

[bb2] Bruker (1998). *SMART* and *SAINT* Bruker AXS Inc., Madison, Wisconsin, USA.

[bb3] Chande, M. S. & Khanwelkar, R. R. (2005). *Tetrahedron Lett.* **46**, 7787–7792.

[bb4] Cremer, D. & Pople, J. A. (1975). *J. Am. Chem. Soc.* **97**, 1354–1358.

[bb5] Davidson, D. & Bernhard, S. A. (1948). *J. Am. Chem. Soc.* **70**, 3426–3428. 10.1021/ja01190a060 18891879

[bb6] Farrugia, L. J. (1997). *J. Appl. Cryst.* **30**, 565.

[bb7] Meldrum, A. N. (1908). *J. Chem. Soc.* **93**, 598–601.

[bb8] Muller, F. L., Constantieux, T. & Rodriguez, J. (2005). *J. Am. Chem. Soc.* **127**, 17176–17177. 10.1021/ja055885z 16332052

[bb9] Ramachary, D. B. & Barbas, C. F. III (2004). *Chem. Eur. J.* **10**, 5323–5331. 10.1002/chem.200400597 15390208

[bb10] Ramachary, D. B., Chowdari, N. S. & Barbas, C. F. III (2003). *Angew. Chem. Int. Ed.* **42**, 4233–4237. 10.1002/anie.200351916 14502744

[bb11] Sheldrick, G. M. (1996). *SADABS* University of Göttingen, Germany.

[bb12] Sheldrick, G. M. (2008). *Acta Cryst.* A**64**, 112–122. 10.1107/S0108767307043930 18156677

[bb13] Spek, A. L. (2009). *Acta Cryst.* D**65**, 148–155. 10.1107/S090744490804362X PMC2631630 19171970

[bb14] Tietze, L. F. & Beifuss, U. (1993). *Angew. Chem. Int. Ed. Engl.* **32**, 131–163.

[bb15] Tietze, L. F., Evers, T. H. & Topken, E. (2001). *Angew. Chem. Int. Ed.* **40**, 903–905. 10.1002/1521-3773(20010302)40:5<903::AID-ANIE903>3.0.CO;2-7 29712158

